# A novel dual-band elliptical ring slot MIMO antenna with orthogonal circular polarization for 5G applications

**DOI:** 10.1016/j.heliyon.2024.e33176

**Published:** 2024-06-15

**Authors:** Swarup Chakraborty, Muhammad Asad Rahman, Md. Azad Hossain, Eisuke Nishiyama, Ichihiko Toyoda

**Affiliations:** a Faculty of Electrical and Computer Engineering, Chittagong University of Engineering and Technology, Chattogram-4349, Bangladesh; b Saga University, 1 Honjo-machi, Saga-shi, Saga, 840-8502, Japan

**Keywords:** 5G, Circular polarization, Dual-band, Elliptical ring slot, MIMO

## Abstract

This paper presents a dual-band 4-element multiple-input-multiple-output (MIMO) antenna featuring orthogonal circular polarization (CP) for Sub-6 GHz 5G applications. The single antenna combines a non-uniform width elliptical ring slot and a feed network, utilizing two L-shaped secondary feed lines to generate CP within the targeted frequency bands (3.55−3.80 GHz and 4.60−4.80 GHz). Using the single antenna as a unit antenna, a 4-element MIMO configuration is devised, employing a mirror technique for element placement to achieve orthogonal CP. This placement method effectively enhances coverage area and enlarges the 3-dB axial ratio bandwidth in the lower band (3.40–3.80 GHz). The common ground connections among elements are established via four strip lines. The antenna shows a good diversity performance considering the envelope correlation coefficient (ECC) result of less than 0.04 and isolation greater than 15 dB. The simulated gains of the MIMO antenna are 4 and 5 dBic in the respective bands. The single and MIMO antennas are fabricated, and the antennas’ performances are evaluated. A good agreement is observed between the measured and simulated results. The dual CP and diversity performances of the MIMO antenna make it more efficient for 5G wireless applications.

## 1 Introduction

Currently, 4G systems are used for telecommunication purposes in most countries. Due to higher speed requirements, fulfilling the user requirement using the 4G systems isn't easy. The 5G systems are becoming handy in solving problems. Some countries like South Korea, the USA, China, and Japan have already taken the initiative to implement the 5G system. There are two possible spectrums for the 5G systems. They are Sub-6 GHz and mm-wave spectrum. The Sub-6 GHz spectrum is becoming preferable as fewer hardware changes are necessary for the initial implementation of the 5G system. Many frequency bands in Sub-6 GHz can be used for 5G applications [1]. For this work, the (3.55–3.8 GHz) and (4.6–4.8 GHz) frequency bands are chosen. The multiple-input-multiple-output (MIMO) antenna is the best candidate for fulfilling the higher data rate requirements within the limited power supply. The MIMO antenna offers more than one antenna that provides multiple channels for transmitting and receiving data. Eventually, the MIMO antenna increases the channel capacity without consuming additional power and frequency bands. A lot of research is going on to make proper single-band and multiband MIMO antennas for 5G communication systems. Nevertheless, multi-band antennas provide more benefits than single-band antennas as they have more bands for operation purposes. In [2], a 3D MIMO antenna system using a magneto-electric dipole antenna as an antenna unit has been proposed. The MIMO antenna has a dual wideband as well as high gain and found its applications in 5G/WiMAX/WLAN/X-Band applications. A four-element MIMO antenna, by placing the elements symmetrically, has been reported for operating in two bands (1.3–1.8, 1.8–2.6 GHz) [3]. A defected ground structure (DGS) is designed to improve isolation; more than 12 dB isolation is achieved in the whole band. A dual-band two-element MIMO system has been designed for the 4G (2.5–2.7 GHz) and 5G (3.4–3.8 GHz) applications [4]. The MIMO antenna consists of two planar inverted-F antenna (PIFA) elements, and the dual-band is realized by designing the slot on the PIFA plate at a minimum current area. A dual-band (operating frequencies are 3.56 GHz and 5.28 GHz) 4-element MIMO antenna has been proposed in [5]. Different sizes of stubs and slots are included in the single radiating element to achieve multiband. Another four-port dielectric resonator MIMO antenna is reported in [6] for sub-6 GHz applications operating at 3.3 and 3.9 GHz. There are also lots of dual-band antennas proposed in [[7], [8], [9]].

The above-described works are linearly polarized (LP) antennas. However, circularly polarized antennas provide extra benefits like multipath distortion, orientation independence, and more coverage area than LP antennas. Many slot antennas are used to produce circular polarization (CP). A microstrip antenna with an arrowhead-shaped slot has been proposed in [10]. By properly adjusting the position of the slot, the CP radiation is achieved. In [11], a triple-band hexagonal slot antenna has been reported. The three CP bands are attained by using three L-shaped slit arms. On the other hand, a microstrip line-fed square-shaped slot antenna has been proposed for dual-band CP operation [12]. A pair of split ring resonators (SRR) is used to sense CP in the upper band. The CP at the lower band is achieved by SRR along with the truncated opposite diagonal corner of the slot. The above-described works have larger size antenna.

Dual-polarized MIMO antennas are gaining popularity as they provide less multipath distortion and transmit twice as much information as single-polarized antennas. Several dual-polarized MIMO antennas have been reported in recent years. A dual-polarized two elements MIMO antenna with an electromagnetic band gap has been proposed in [13]. The LP antenna has 0° and 90° polarizations. A two-element ultra-wideband MIMO antenna with both LP and CP has been demonstrated, and the CP is found by etching an I-shaped strip line on the hexagonal slot of every single antenna [14]. A dual LP MIMO antenna has been proposed for 5G smartphone applications [15]. Two different shaped (C-shaped coupled feed and L-shaped monopole slot) arrays are used to find orthogonal polarizations. In the above-described research works, a maximum of them are LP antennas. A 2-element and 4-element dual CP MIMO antennas have been proposed in [16,17], respectively, yet both are single-band MIMO antennas. Another single band 4-element MIMO antenna has been reported in [18], where an L-shaped feed line and the DGS are used to achieve CP in the desired band. Again, in [19], the authors of this paper have proposed a four elements orthogonal CP single band MIMO antenna for 5G application in the frequency band (3.4–3.8 GHz), and two secondary feed lines in the unit antenna are used to generate CP in the desired band.

This article proposes a dual-band four-element orthogonal CP MIMO antenna for 5G applications. The operating bands of the MIMO antenna are 3.55–3.8 GHz and 4.6–4.8 GHz. The proposed antenna is designed based on the single-band CP MIMO antenna proposed in [19] and the dual-band MIMO antenna reported in [20]. In the proposed antenna, two CP frequency bands are achieved by modifying the feed structure used in the single-band CP MIMO antenna. On the other hand, the dual-band MIMO of [20] has two antenna elements placed side by side without applying polarization diversity between the elements. As a result, the AR of this MIMO is less satisfactory. However, the limitation of the antenna has been overcome in the proposed MIMO antenna by applying polarization diversity between neighboring elements. Initially, a microstrip line-fed slot antenna was designed where the slot had a non-uniform width and was elliptical ring-shaped. The feed line is created using the unequal feed lines technique, where two L-shaped strip lines of different lengths are used to generate CP. The MIMO antenna is a combination of four single-slot antenna elements. All the elements are placed, so the polarization of the side-by-side elements is opposite, and the polarization of the diagonal elements is similar. Due to this type of orientation, every element is surrounded by opposite polarized elements in the MIMO organization. Hence, the MIMO antenna has dual CP, which helps the antenna to increase the broadcasting area by utilizing both front and back radiation. Additionally, the antenna provides good isolation among the antenna elements. The diversity performance of the antenna is satisfactory for MIMO operation for 5G cellular applications. Both single and MIMO antennas show satisfactory agreement between measured and simulated performances.

The article has two main sections. The first section discusses the theoretical background of an elliptical ring slot antenna. Then, the design and performance of the proposed elliptical ring slot antenna are covered in the second section.

## 2 Elliptical ring slot

Slot antennas provide front and back radiation with orthogonal polarization. As a result, it is beneficial for the MIMO antennas to increase the coverage area [21]. Again, the ellipse has two independent variables (major and minor axis) compared to one variable (radius) in the circle. It is noticed that it is more flexible to design an elliptical antenna where the system's overall size is restricted. Therefore, the elliptical slot antenna is more beneficial for the MIMO antenna in overcoming the size limitation. There is a lot of research on elliptical-shaped patch antennas, and [22,23] have discussed the characteristics of the elliptical-shaped antenna. Though there are various elliptical ring slot antennas like [24], a little work on the behavior of the elliptical ring slot antenna is found. Hence, the theoretical analysis of the ring slot antenna is briefly discussed in this section.

The fundamental mode of the elliptical ring slot antenna usually depends on the slot's perimeter. The perimeter should equal approximately one wavelength of the desired fundamental frequency. To calculate the value of either the major axis or minor axis for the specific wavelength, the following equations are used to design an elliptical ring slot antenna. Note that the equations provide approximate values.

(1) $$C=\lambda =2\pi \sqrt{\frac{a^{2}+b^{2}\;}{2}}$$

(2) $$\text{then},b=\sqrt{\frac{\lambda^{2}}{2\pi^{2}}-a^{2}}\;;\;\frac{\lambda^{2}}{2\pi^{2}}>a^{2}$$

Where *a* is the length of the major axis, *b* is the length of the minor axis, λ is the wavelength, and *C* is the outer perimeter of the ring slot.

**Fig. 1:**
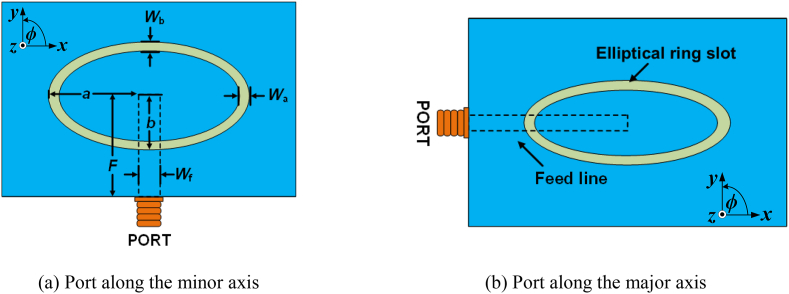
Elliptical ring slot antennas where *a* = 9.6 mm, *b* = 7 mm, *W*_a_ = *W*_b_ = 1 mm, *F* = 15 mm, *W*_f_ = 1.44 mm.

Fig. 1 shows an elliptical ring slot antenna with two different excitation positions for 3.7 GHz. The 0.8-mm thick FR4 ($\epsilon_{r}$ = 4.4) substrate is used and λ equals 44.0 mm. By taking the perimeter of the ring slot equal to the ***λ***, the major axis length, *a*, and minor axis length, *b*, are calculated. Initially, *a* is chosen to be 9 mm, and then the minor axis length, *b* = 4.13 mm, is calculated using equations (1), (2). During simulation, the exact values of *a* and *b* are 9.7 mm and 7 mm, respectively, corresponding to the perimeter of 53.14 mm.

**Fig. 2:**
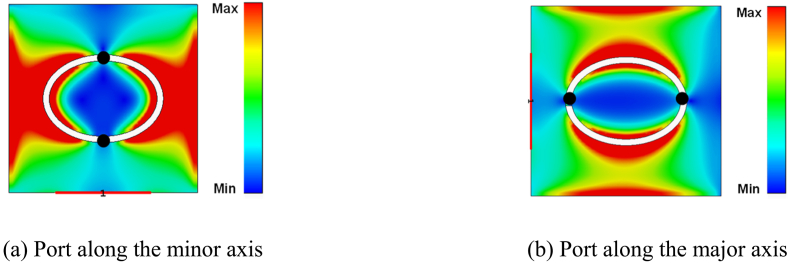
Surface current distribution of an elliptical ring slot antenna at 3.7 GHz. The black dot points represent the current null points.

**Fig. 3:**
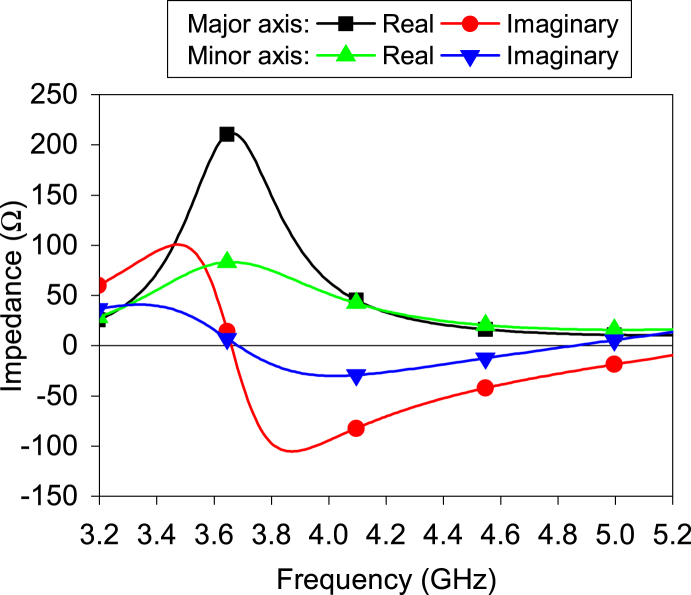
Antenna input impedances for placing the port at the major and minor axes.

Fig. 2 displays the surface current distribution of the ring slot antenna at 3.7 GHz. Two null points (highlighted by dark circles) are present in both cases. Hence, the antenna resonates at the fundamental frequency in both port configurations [25].

a. Impedance matching:

The elliptical ring slot has different lengths concerning the major and minor axes. Hence, the antenna impedance varies between the axes. The port was placed individually along both axes during the simulation, as shown in Fig. 1, to check the antenna impedance. Fig. 3 illustrates the antenna impedance for both port locations (along the minor and major axes). The imaginary part of the impedance is equal to zero at 3.7 GHz in both cases. For both the major and minor axes, the imaginary part is in the inductive region before the resonance point and in the capacitive region after the resonance point. The imaginary value for the major axis is greater than the minor axis in both the inductive and capacitive regions. Similar performance is achieved for the real part also. It is evident that real impedance has a relatively lower value when the port is placed along the minor axis, as the minor axis has a shorter length than the major axis. Hence, only this configuration is used for further study.

**Fig. 4:**
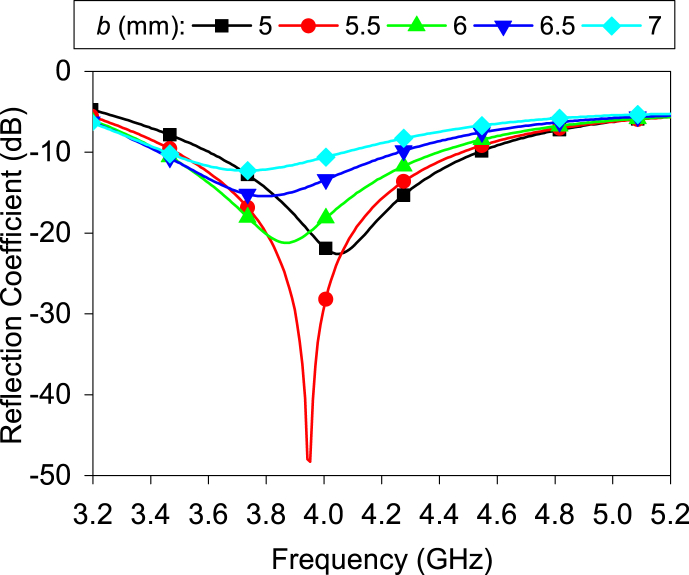
Effect of the minor axis length (*b*) on the reflection coefficient.

Fig. 4 shows the slot antenna's reflection coefficient for different minor axis lengths. It is noticed that reducing the minor axis length of the slot without changing its circumstances makes proper impedance matching possible. The minimum reflection coefficient is found at *b* = 5.5 mm. The reason can be explained by the antenna's real impedance result.

**Fig. 5:**
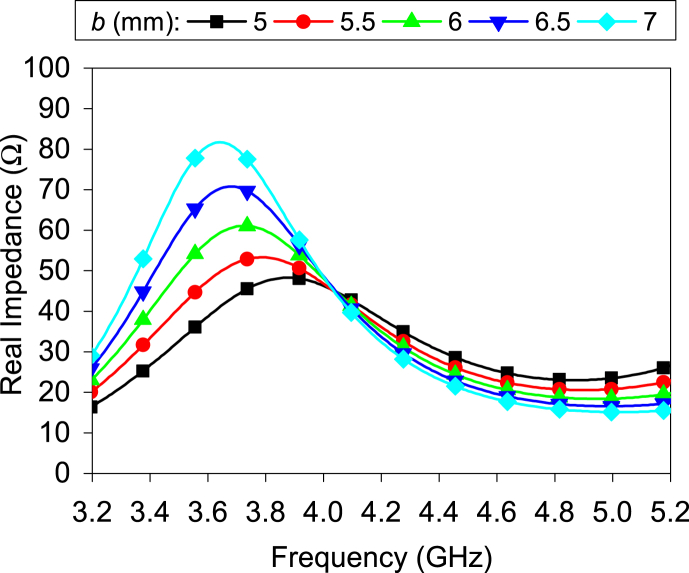
Real impedance for different value of *b*.

**Fig. 6:**
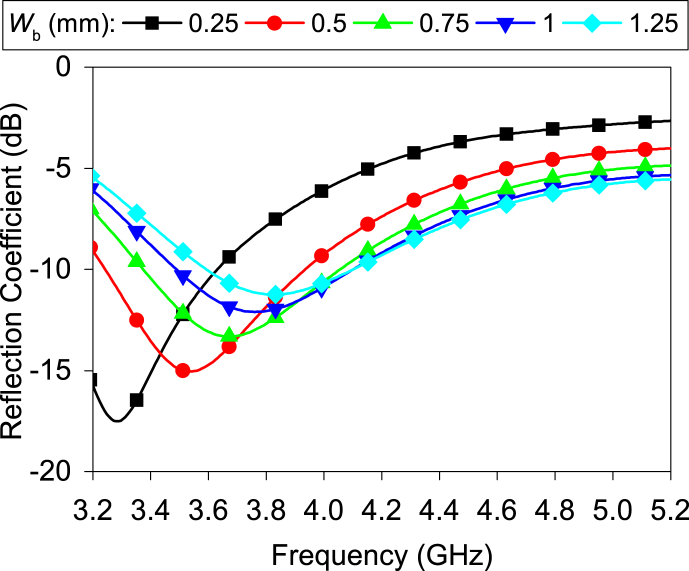
Effect of the slot width (*W*_b_) on reflection coefficient.

Fig. 5 displays the real impedance of the antenna. It is seen that the real impedance is proportional to the length of the minor axis when the perimeter is constant. When *b* = 5.5 mm, the antenna's real impedance is approximately matched to the port impedance (50 Ω). During the simulation, the slot width is uniform. From Fig. 4, it is evident that parameter *b* significantly affects the antenna's bandwidth. The 10-dB impedance bandwidth reduces when *b* is more than 6 mm. The reason can be explained in Fig. 5; the real impedances increase significantly at the resonance frequency when *b* is more than 6 mm.

b. Effect of the unequal slot:

The unequal slot has a noticeable effect on impedance matching. Fig. 6 illustrates the impact of the slot width (*W*_b_) along the minor axis on the impedance matching when the slot width (*W*_a_) along the major axis remains fixed. The real impedance is decreased (from approximately 90 Ω–20 Ω) when *W*_b_ is reduced; thus, the reflection coefficient improves. A similar effect is also found for slot width along the major axis. From the above discussion, using this technique, it is possible to design the elliptical ring slot in any limited size. Furthermore, the 10-dB impedance bandwidth is inversely propositional to the width (*W*_b_). The bandwidth reduces by around 100 MHz for each variation (0.5 mm ≤ *W*_b_ ≤ 1.25 mm).

## 3 Proposed MIMO antenna using elliptical ring slot

a. Single antenna design

**Fig. 7:**
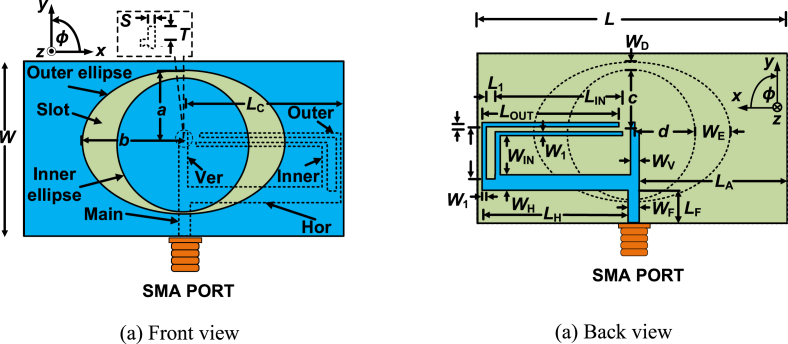
Complete view of the single antenna.

Fig. 7 shows the overall view of the single antenna. The total size of the proposed single antenna is 64 × 34 mm^2^. Initially, two ellipses are designed in the rectangular-shaped ground plane to create the ring slot. The outside ellipse, denoted as the “Outer ellipse,” has a major axis length of *a* and a minor axis length of *b*. The inside ellipse, having a major axis length of *c* and a minor axis length of *d*, is called an “Inner ellipse”. The center of the “Inner ellipse” is the center point of the overall antenna structure. To create an unequal width slot, the center point of the “Outer ellipse” is moved by 0.5 mm along the positive *y*-axis and again is shifted to a distance of 0.5 mm along the negative *x*-axis. The antenna is excited by a feed line denoted as “Main”. First of all, the “Main” feed line is divided into two secondary feed lines. One of them is a horizontal feed line denoted as “Hor” situated along the *x*-axis. Another feed line is a vertical feed line called “Ver,” located along the *y*-axis. The “Hor” feed line is again segmented into two L-shaped feed lines to attain multi-band.

**Fig. 8:**
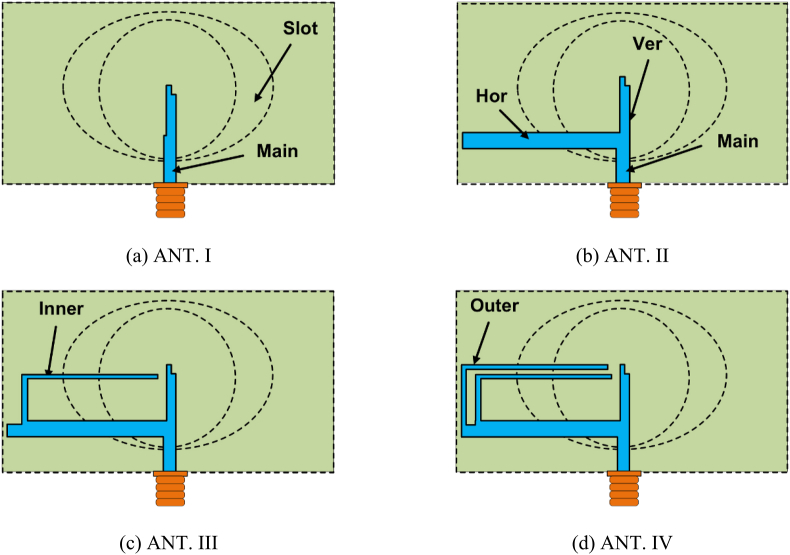
Design stages of the single antenna.

**Fig. 9:**
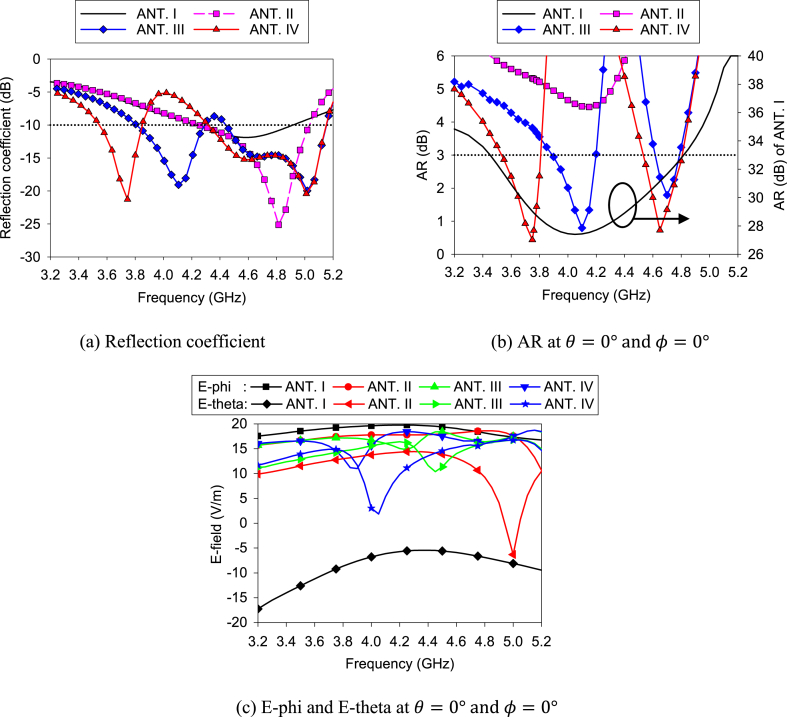
Performances of the different design states.

Fig. 8 depicts the four states of single antenna design. Again, Fig. 9 shows the reflection coefficient, AR, and E-fields of the antenna design states. ANT. I consists of a non-uniform width elliptical ring slot on the ground plane and a vertical feed line on the substrate's other side. Fig. 9a shows the reflection coefficient of all the antenna states. ANT. I is an LP antenna with an AR of more than 20 dB. The antenna has only one feed line. As a result, the antenna has a high E-phi, which is understandable from Fig. 9c. The antenna has a resonance at 4.6 GHz with a bandwidth of 700 MHz (corresponding percentage bandwidth = 15.2%). To improve impedance matching and AR, the “Main” feed line is segmented into two feed lines (“Hor” and “Ver” feed lines) in the ANT. II. ANT. II has improved impedance matching with nearly the same bandwidth as the ANT. I. Due to the feed line (“Hor”), the AR is reduced to 4 dB, as shown in Fig. 9b. The “Hor” feed line is situated along the *x*-axis. As a result, it empowers E-theta. Hence, the ANT. II has two improved E-fields (E-phi and E-theta), and AR is decreasing. To reduce the AR further, ANT. III adds an L-shaped feed line (Inner) with the “Hor” feed line.

To achieve CP, two orthogonal E-fields of equal magnitude are needed. Due to the “Inner” feed line, both E-fields have approximately the same magnitude in 4.1, 4.28, 4.7, and 4.9 GHz, as shown in Fig. 9c. However, it is noticed that the angle between the E-fields is nearly 90° (85.69° and 97.2°.) only at 4.1 and 4.7 GHz. Therefore, in both frequencies, the conditions of the CP are fulfilled, and 3-dB AR bandwidths are achieved. Both frequencies are allowed for 5G applications, and the antenna works as a CP antenna in both bands. Conversely, at 4.28 and 4.9 GHz, the angle between the E-fields is 103.9° and 236°, respectively. As a result, CP conditions are not fulfilled, and no 3-dB AR bandwidth is attained in that frequency. Moreover, adding the “Inner” feed line, a lower resonance at 4.1 GHz has developed in the ANT. III. To achieve resonance at 3.7 GHz, as it is the more popular allocated frequency for 5G applications, another L-shaped feed line (“Outer”) is inserted with the horizontal feed line (“Hor”) in the ANT. IV. By inserting the “Outer” feed line, both impedance and AR bands of 4.1 GHz are moved to the lower frequency (3.6–3.8 GHz) band, which is another interesting band for this work. The magnitude difference between the E-fields becomes larger at 4.2 GHz. Therefore, AR is increased by more than 3 dB in that frequency. On the other hand, the magnitude difference between two E-fields at 3.7 GHz is decreased, and the angle between both E-fields is approximately 270°. As a result, AR is reduced at 3.7 GHz. Again, the angle between the E-fields is changed to approximately 270° in 4.65 GHz. Hence, overall, the CP performance is improved at the final design state, which is evident from Fig. 9b.

**Fig. 10:**
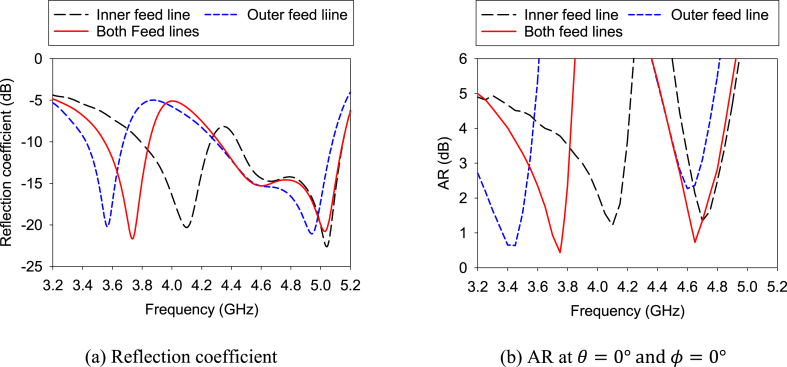
Effect of the L-shaped feed lines on the single antenna.

Fig. 10 illustrates the effect of L-shaped feed lines (“Inner” and “Outer”) on reflection coefficient and AR. The figure shows that the “Inner” feed line has created two resonance points. Again, the “Outer” feed line generates two resonance points while the higher resonance point is the same as that is created by the “Inner” feed line. But the other tuned point is found to have a lower frequency than that of the “Inner” feed line. When both feed lines are inserted at a time, a lower resonance is found in the middle of the previous lower resonances created by the feed lines, and a lower resonance is found in the middle of the previous lower resonances created by the feed lines individually.

**Fig. 11:**
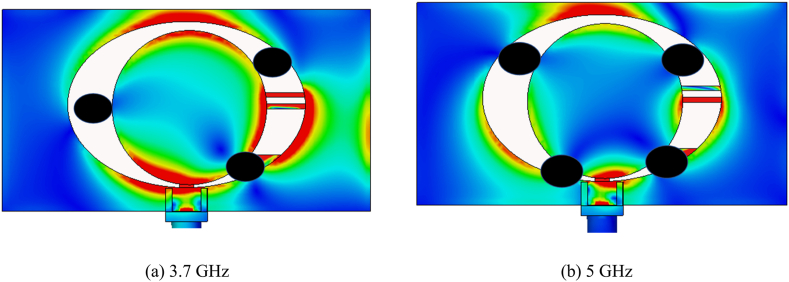
Surface current distribution of the single antenna. The black dot points represent the current null points.

From the surface current distribution, finding the resonance points of the dual-band antenna is possible. Fig. 11 shows the surface current distribution at 3.65 GHz. Generally, one wavelength has two null points. However, in Fig. 13a, it is evident that there are three null points. Therefore, the antenna is resonating at one and a half wavelength at 3.65 GHz. Again, we see four null points at 4.65 GHz in Fig. 11b. The antenna is tuned at two wavelengths at 4.65 GHz. Table 1 is populated by the final value of all parameters used to design the single antenna.

b. MIMO antenna design

**Table 1:** The numerical value of the design parameters.

Parameters	***a***	***b***	***c***	***d***	***L***	***L***_**A**_	***L***_**c**_	***L***_**F**_	***L***_**H**_	***L***_**in**_	***L***_**out**_	***L***_**1**_
Value (mm)	20	14	13	13	62	29.8	30.5	6.4	29.1	25.4	27.3	1.5

Parameters	*S*	*T*	*W*	*W*_D_	*W*_E_	*W*_F_	*W*_H_	*W*_in_	*W*_out_	*W*_V_	*W*_1_	*W*_2_

Value (mm)	0.9	1.8	34	1.4	7	2.4	3.1	7.8	10.5	1.7	0.9	0.75

**Fig. 12:**
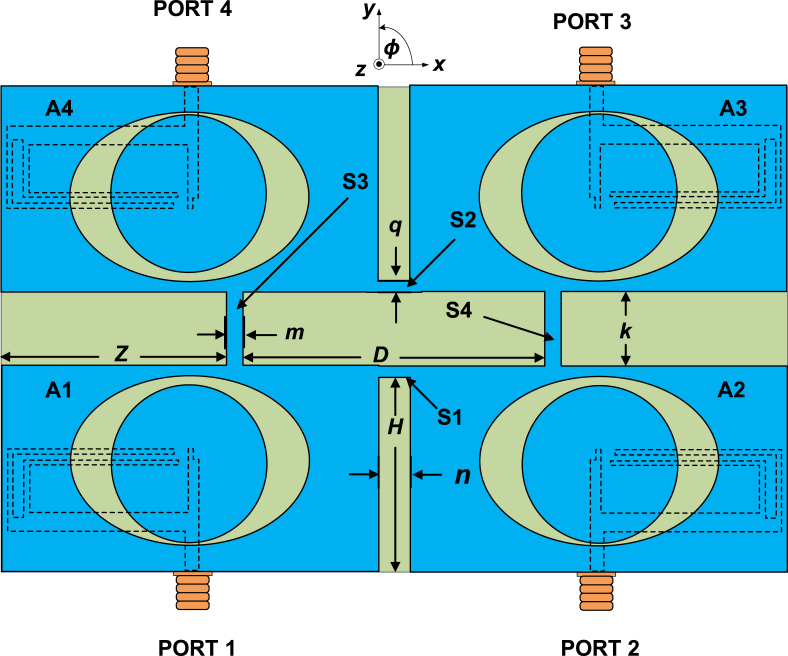
The overall view of the MIMO antenna. The parameters (in mm): *n* = 3.0, *q* = 1.0, m = 1.0, *k* = 12.5, *D* = 50, *H* = 33, and Z = 37.5.

Fig. 12 shows the overall view of the MIMO antenna. The MIMO antenna has four single-slot antennas denoted as A1, A2, A3, and A4. In the beginning, two single-ring slot antennas (A1 and A2) are placed side by side at a distance of *n* mm along the *x-*axis. To achieve polarization diversity, the antenna element A2 mirrors A1. It is noted that there is no entire ground plane between the antenna elements because it reduces the performance of the MIMO antenna. The effect on the whole ground will be discussed later. A single strip (“S1”) of width of *n* and height of *q* has been inserted between the antenna elements to maintain common ground between the antenna elements. A mirror of similar types of antenna structure (A3 and A4) is inserted along the *y*-axis at a distance of *k*. Two single strip lines (height *k* and width *m*) denoted “S3” and “S4”, respectively, are designed along the *y*-axis on the ground plane to reduce the interference among the antenna elements. The antenna is designed on the Teflon substrate, sandwiched between two 0.018 mm thick copper layers. The substrate's thickness and dielectric constant are 0.8 mm and 2.15, respectively.

**Fig. 13:**
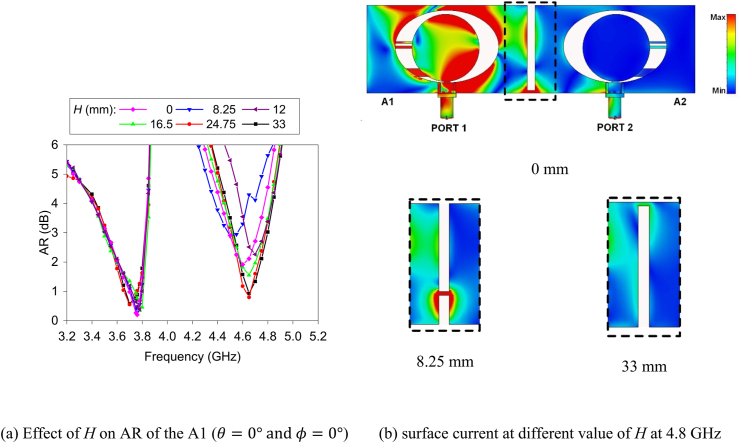
Influence of the position (*H*) of the "S1" on AR of the MIMO antenna.

Position (*H*) of the strip line “S1” greatly affects the AR of the higher band. Fig. 13 shows the impact of the position (*H*) of the “S1” on the AR of the antenna element A1. It is noticed from the figure that a better result is found when *H* is more significant than 16.5 mm. In fact, at *H* = 8.25 mm, the worst outcome is attained. The reason can be explained by the surface current distribution of the strip line at different positions (*H*) of “S1”. Fig. 13b illustrates the surface current distribution at 4.8 GHz at *H* = 0, 8.25, and 33 mm when PORT 1 is excited. There is a low current flow between the antenna when *H* = 33 mm, and hence, there is low surface wave propagation between the antenna elements. Thus, an enhanced outcome is found. On the other hand, high current flow appears when *H* is equal to 0 mm and 8.75 mm. These high current flows increase the difference between the electric components (E-theta and E-phi) of the A1 due to high interference, and eventually, the corresponding results of AR are degraded.

**Fig. 14:**
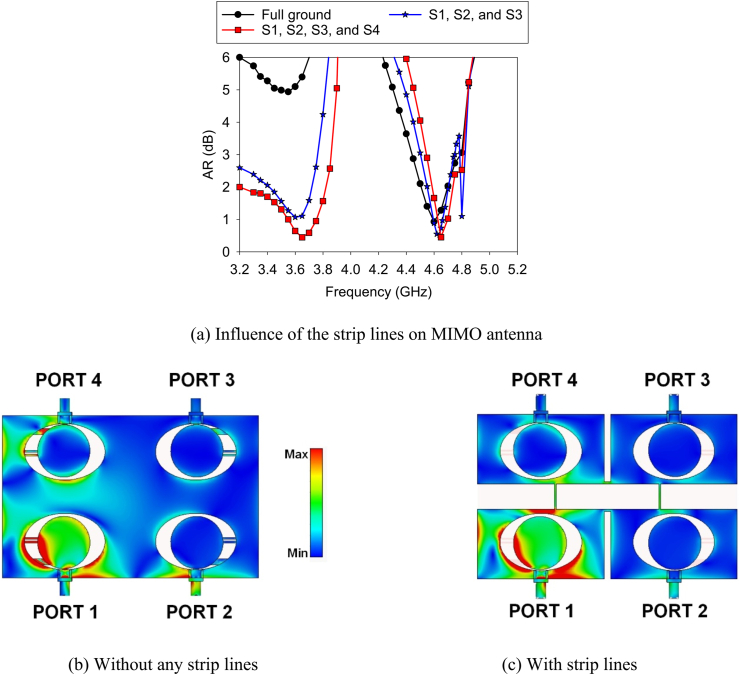
Common ground effect on the MIMO antenna.

A plane ground is initially designed between the antenna elements (A1, A2) and antenna elements (A3, A4). Fig. 14 displays the impact of the full ground and strips on the AR of the A1. At full ground, the AR at the lower band is greater than 4 dB. After inserting strip line “S4”, the AR is reduced, but the result is more than 1 dB. Finally, adding another strip line S3 improves the AR at the lower band, and less than 0.5 dB is achieved. The reason for the degradation of AR can be clarified from the surface current distribution. At complete ground condition, there is a current flow among the antenna elements, especially in the A4 when PORT 1 is excited. This creates more interference among the antenna elements, ultimately degrading AR. The amount of current is diminished by inserting the strip lines as the strip line (“S3”) provides a high impedance between A1 and A4. Hence, better AR result is found. A similar coincidence happened between the A2 and A4 for the strip line (“S4”).

c. Improve AR Bandwidth at the MIMO antenna

**Fig. 15:**
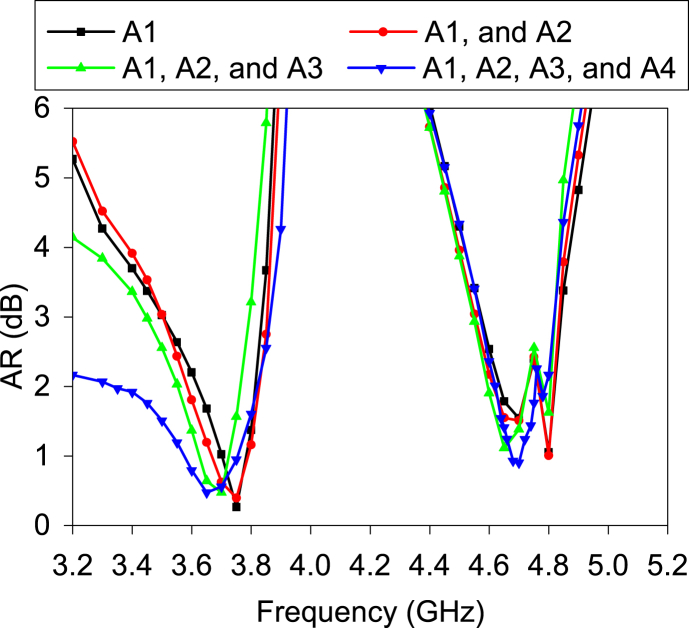
Impact of the antenna elements on the AR of A1 at *θ* = 0° and ∅ = 0°.

**Fig. 16:**
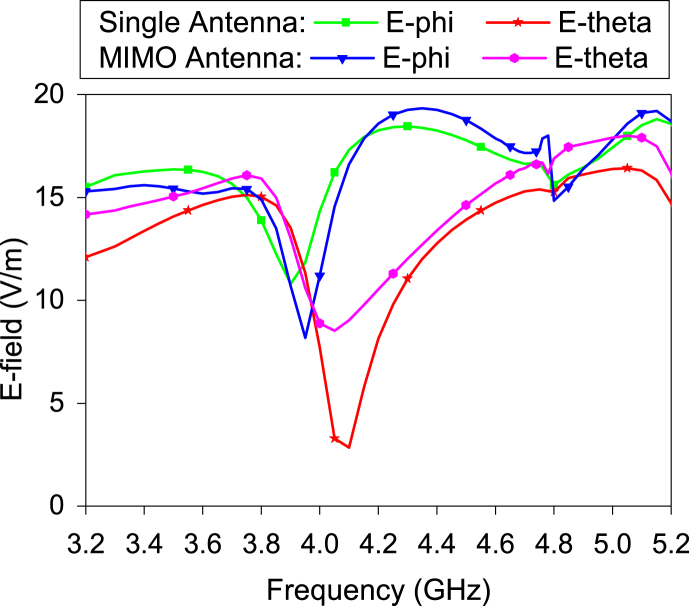
E-field of MIMO antenna and single antenna at *θ* = 0° and ∅ = 0°.

It is noticed that increasing the number of elements, AR is also increased. Fig. 15 shows the AR variation of the A1 with the number and placement of the antenna elements. There is no effect on AR when two antenna elements are placed. After introducing the third antenna element (A1), the AR of the A1 is slightly reduced. Again, after introducing the fourth element (A4), the AR is significantly abridged in the lower frequency range. The reason can be understood from the following figure. Fig. 16 exhibits the E-field variation between the single and MIMO antenna. Both E-fields are affected by the MIMO arrangement. The E-theta component is increased in both bands. The E-phi component is reduced in the lower band but increased in the higher band. Hence, the distance between both E-fields is reduced in a lower band in the MIMO antenna and remains the same distance in the higher band as much as the distance in the single antenna. Therefore, the 3-dB AR bandwidth of the MIMO antenna at a lower frequency band is increased.

## 4 Performance of the MIMO antenna

**Fig. 17:**
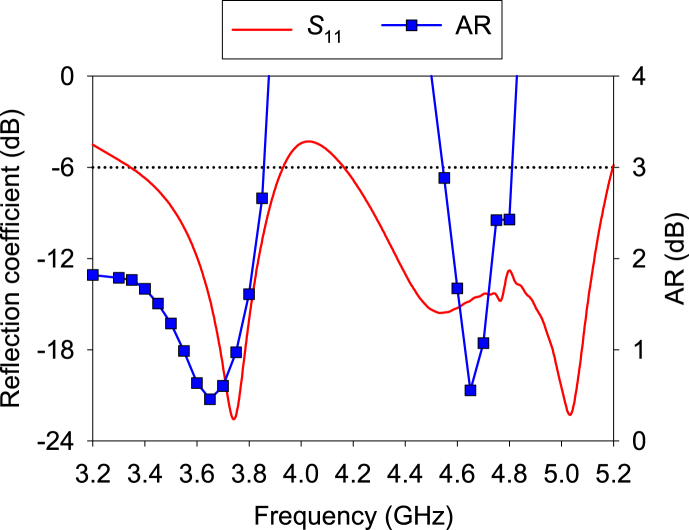
Simulated *S*_11_ and AR (∅ = 0°and θ = 0°) of the proposed MIMO antenna.

Fig. 17 exhibits the *S*_11_ and AR of the A1. It is noted that similar results are found for other antenna elements also. For brevity, only the result of the A1 is discussed. Two resonance frequencies are achieved for both *S*_11_ and AR. The 10-dB impedance bandwidths are 3.55–3.86 GHz and 4.3–5.15 GHz, corresponding to percentage bandwidths of 8.5% and 18.3%, respectively. Moreover, the 3-dB AR fraction bandwidths are 17.8% (3.2–3.85 GHz) and 5.6% (4.54–4.8 GHz). Therefore, the antenna covers 3.55–3.8 and 4.6–4.8 GHz frequency bands, as shown in the figure. Again, 3-dB AR bandwidths are found in both bands. AR is achieved less than 0.5 dB in the interested bands.

**Fig. 18:**
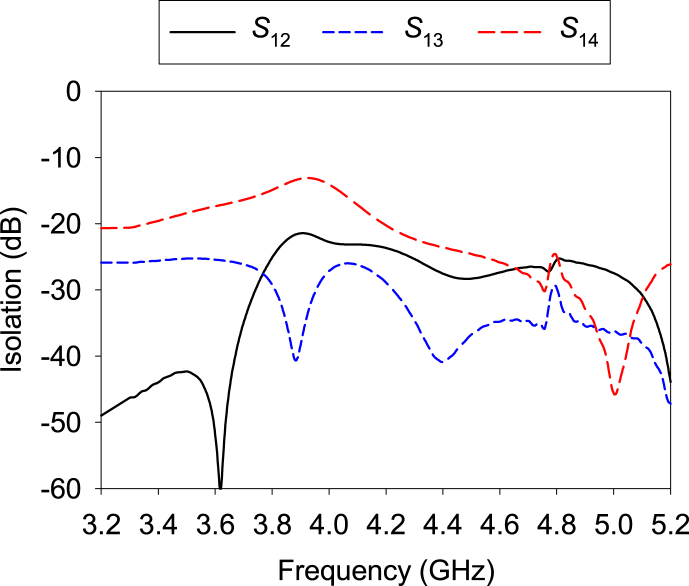
Simulated isolation of the proposed MIMO antenna.

Fig. 18 shows the proposed MIMO antenna's isolation. At the lower band, maximum isolation is more than 50 dB for *S*_12_, and minimum isolation is 15 dB for *S*_14_. The isolation of different elements at the higher band ranges from 20 to 45 dB. Therefore, excellent isolation is achieved in both bands.

**Fig. 19:**
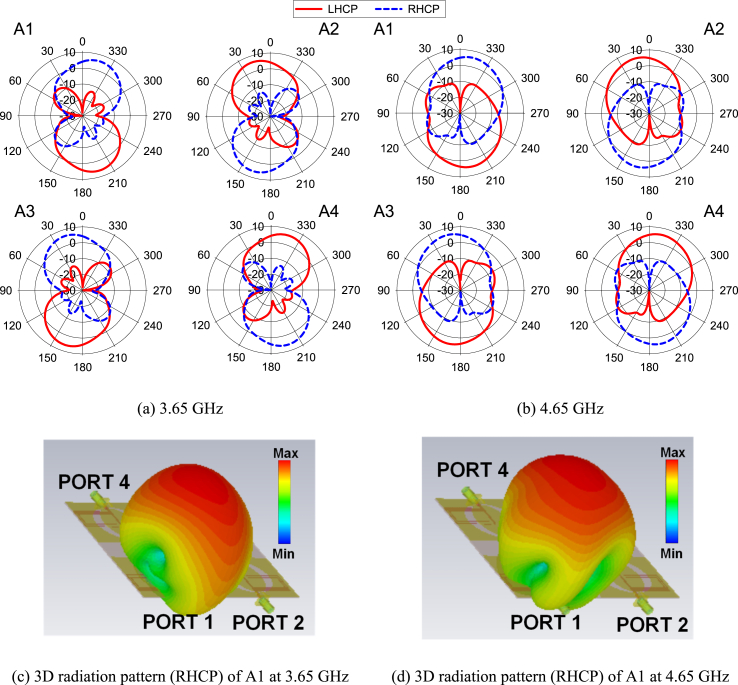
Radiation pattern of the proposed MIMO antenna at *xz*-plane.

Fig. 19a and b shows the radiation pattern of the different elements in the *xz*-plane at 3.65 GHz and 4.65 GHz. It is noted that a similar radiation pattern is also achieved in the *yz*-plane. For brevity, only the radiation patterns in the *xz*-plane are discussed. The polarization of the broadside of A1 and A3 is RHCP, while LHCP is found for A2 and A4. The cross-polarization suppression is achieved by more than 30 dB at both frequencies for every element. The main lobe of every element shows strong radiation performance with a gain of 4 dBic at *θ* = 0° at 3.65 GHz and around 5 dBic at *θ* = 0° at 4.65 GHz. Fig. 19c and d illustrate the 3D radiation patterns at both bands when PORT 1 is excited.

Fig. 19 shows that the front and back radiations of the A1 are RHCP and LHCP in both frequencies, respectively. Again, the A2 has both polarizations (LHCP and RHCP) in reverse directions of A1. Similar patterns are achieved for other elements (A3 and A4). As a result, the MIMO antenna can achieve both radiation (LHCP and RHCP) by utilizing the front and back sides of the antenna. This characteristic of the MIMO antenna increases the coverage area and offers extra benefits for receiving and transmitting data.

**Fig. 20:**
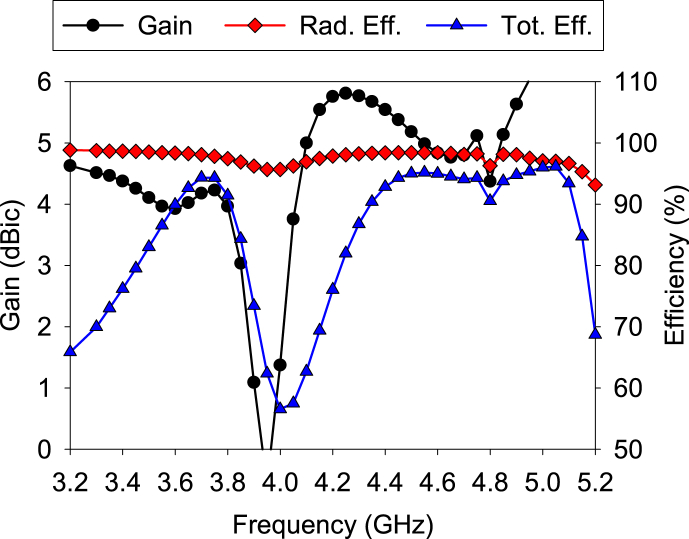
Gain and efficiencies.

Fig. 20 displays the gain and efficiencies of the A1. The results of the gains and efficiencies of the other elements are similar to those of the A1. Both radiation and total efficiencies are found to be more than 90% in all the bands. The gains at lower and higher bands are more than 4 dBic and nearly uniform in the bands of interest.

Envelope correlation coefficient (ECC) performance is evaluated to analyze the diversity performance of the MIMO antenna. The ECC values describe how much the antenna elements work independently. The acceptable limit of ECC is less than 0.05 [20].

**Fig. 21:**
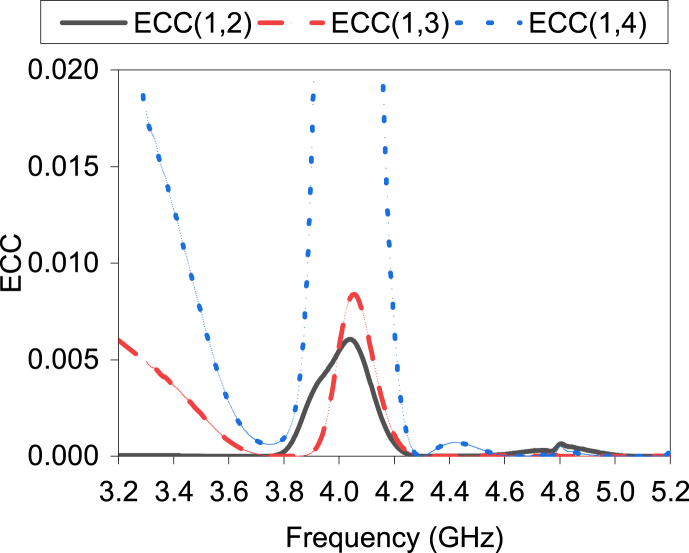
ECC.

Fig. 21 shows the MIMO antenna's ECC parameters. All ECCs are less than 0.02 in the interest bands. Considering the ECC, the antenna's diversity performances are promising.

## 5 Antenna's fabrication and measured performance

**Fig. 22:**
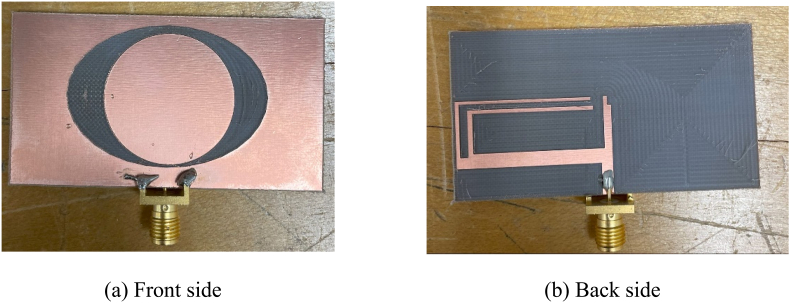
Prototype of the single antenna.

**Fig. 23:**
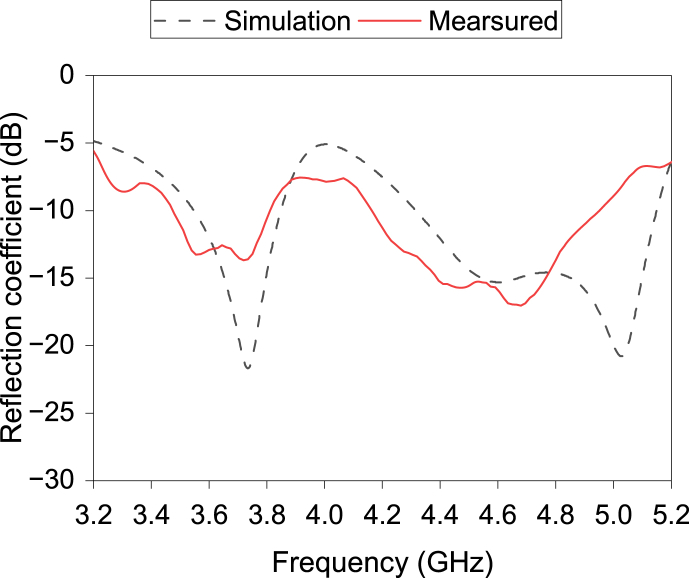
Simulated and measured reflection coefficient of the single antenna.

Fig. 22 displays the fabricated prototype of the single antenna. Fig. 23 plots the proposed antenna's practical and simulated reflection coefficient. The keysight PNA N52227A has been used for the S-parameter measurement. Both outcomes have two resonance frequencies. The simulated 10-dB impedance bandwidths are 8.2% and 18.0% at the respected resonance frequencies, while the measured respected bandwidths are 9% and 16.8%. A good agreement is found between the measured and simulated outcomes.

**Fig. 24:**
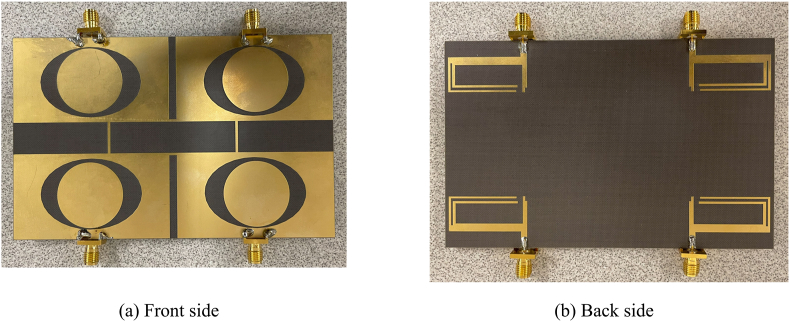
Prototype of the MIMO antenna.

**Fig. 25:**
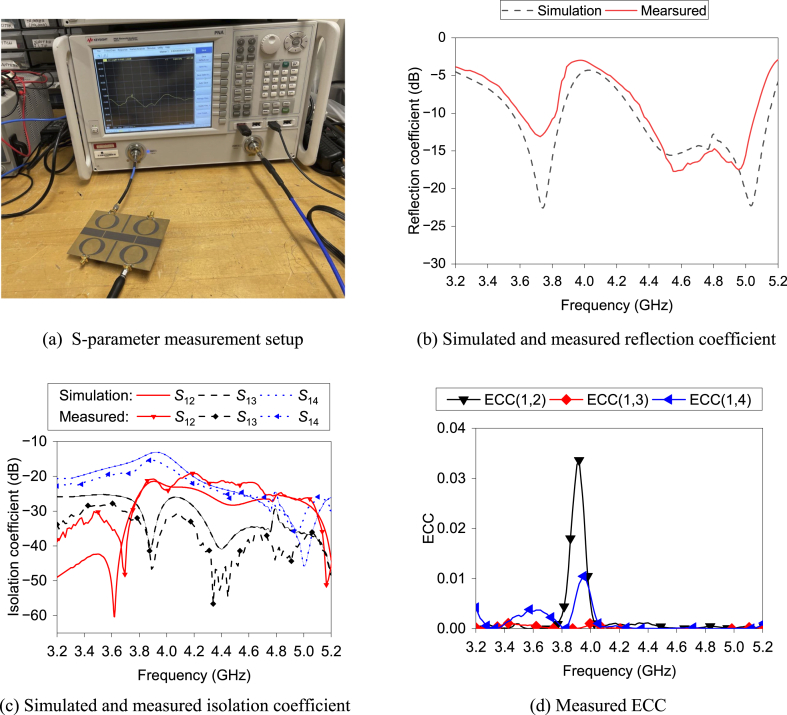
S-parameters and ECC of the proposed MIMO antenna.

Fig. 24 shows the fabricated proposed MIMO antenna. Fig. 25 (a) and (b) display the S-parameter measurement setup and the simulated and practical *S*_11_ result of the MIMO antenna, respectively. The measured 10-dB bandwidths are 6% and 15.2% at the respective frequency bands, while the simulated fractional bandwidth is found to be 8.2% and 18.3%. There are deviations between the simulation and measured results. The anomalies may be the result of fabrication and measurement issues. The experimental results cover 3.6–3.8 and 4.6–4.8 GHz frequency bands.

Fig. 25c illustrates both simulated and measured isolation between the antenna elements. All the results are less than −15 dB in both measurement and simulations. Therefore, good isolation is achieved among the antenna elements in the fabricated antenna. Fig. 25d displays the measured ECC results between the antenna elements. All ECC results are less than 0.05, which is the acceptable ECC limit.

Table 2 compares the MIMO antennas and the proposed MIMO antenna. Ref. [5,6] have reported the multi-band MIMO antennas. But the polarization of all the antennas is LP. Ref. [14,16,17] reported polarization diversity single band MIMO antennas where the antenna of Ref. [14] has both LP and CP. Ref. [18] proposed a 4-element UWB CP antenna where 3-dB AR bandwidth is achieved in 4.8–6.9 GHz, but polarization diversity is absent in this antenna. Only Ref. [16,17,19], and [26] have dual CP, though they are not multi-band antennas. This work proposes a 4-element dual-band MIMO antenna. The antenna has orthogonal CP (LHCP and RHCP). ECC delineates the proposed MIMO antenna's promising diversity performance.

**Table 2:** Comparison with other MIMO antennas.

Ref.	Antenna Elements	LP/CP	10-dB Imp. BW (%)	Isolation dB (>)	3-dB AR BW (%)	Gain (dBi/dBic)	ECC (<)
[5]	4	LP	1.82, 2.8	22	–	4.2, 2.8	0.04
[6]	4	LP	18.7, 14.6	20	–	5.8, 6.2	0.04
[14]	2	LP, CP	112.1	17	34.4	4.91	0.15
[16]	2	Dual CP	9.2	33	1.5	5.34	2.6 × 10^−2^
[17]	4	Dual CP	7.1	19.37	6.3	7.95	2.45 × 10^−4^
[18]	4	CP	65.0	15	20.1	–	0.01
[19]	4	Dual CP	10.8	20	13.5	5	0.004
[26]	2	LP, Dual CP	50	11.75	45.7	1.078	0.02
[27]	2	CP	9.3	28	11.1	8.0	–
**This Work**	4	Dual CP	6, 15.2	15	*17.8, *5.6	*4, *5	0.04

(*)-simulated data.

## 6 Conclusion

In this communication, a dual-band orthogonal CP MIMO antenna is proposed. The MIMO antenna consists of four single-ring slot antennas. The slot of the single antenna is non-uniform width elliptical ring-shaped. An unequal feed line is used to excite the single antenna, where branches of the main feed line are used to generate CP in both bands. The single antenna elements are positioned mirror-symmetrically in a MIMO arrangement. Because of the mirror, the MIMO antenna has orthogonal CP in side-by-side antenna elements. Due to dual CP, the coverage area of the MIMO antenna is improved. Moreover, the antenna shows a promising MIMO diversity performance, considering the ECC results of the MIMO antenna. All the outcomes of the MIMO antenna prepare the antenna more suitable for 5G cellular applications in 3.6–3.8 GHz and 4.6–4.8 GHz frequency bands.

## Data availability statement

No data was used for the research described in the article. All the simulated and measured data of the antenna's may be provided by the authors upon valid request.

## CRediT authorship contribution statement

**Swarup Chakraborty:** Writing – review & editing, Writing – original draft, Visualization, Validation, Software, Investigation, Formal analysis, Conceptualization. **Muhammad Asad Rahman:** Writing – review & editing, Visualization, Supervision, Methodology, Conceptualization. **Md. Azad Hossain:** Writing – review & editing, Conceptualization. **Eisuke Nishiyama:** Writing – review & editing. **Ichihiko Toyoda:** Writing – review & editing.

## Declaration of generative AI and AI-assisted technologies in the writing process

During the preparation of this work the authors used ChatGPT and Grammarly in order to improve the language and readability. After using this tool, the authors reviewed and edited the content as needed and took full responsibility for the content of the publication.

## Declaration of competing interest

The authors declare that they have no known competing financial interests or personal relationships that could have appeared to influence the work reported in this paper.
